# Urinary Clusterin Is Upregulated in Nephropathia Epidemica

**DOI:** 10.1155/2018/8658507

**Published:** 2018-02-26

**Authors:** Ekaterina V. Martynova, Adelya N. Maksudova, Venera G. Shakirova, Sayar R. Abdulkhakov, Ilsiyar M. Khaertynova, Vladimir A. Anokhin, Vilena V. Ivanova, Ilesanmi M. Abiola, Ekaterina E. Garanina, Leisan G. Tazetdinova, Aigul H. Valiullina, Svetlana F. Khaiboullina

**Affiliations:** ^1^Institute of Fundamental Medicine and Biology, Kazan Federal University, Kazan, Republic of Tatarstan, Russia; ^2^Kazan State Medical University, Kazan, Republic of Tatarstan, Russia; ^3^Kazan State Medical Academy, Kazan, Republic of Tatarstan, Russia; ^4^University of Nevada, Reno, NV, USA

## Abstract

Kidney insufficiency is a hallmark of nephropathia epidemica (NE). Little is known about the mechanisms of the NE kidney pathology, with current knowledge mainly based on findings in postmortem tissue. We have analyzed kidney damage biomarkers in urine collected from early- and late-phase NE using Bio-Plex kidney toxicity panels 1 and 2. To determine the disease specificity, kidney damage biomarkers were also analyzed in urine samples from patients diagnosed with gout, type 2 diabetes, systemic lupus erythematosus, and chronic kidney insufficiency. Analysis of 12 biomarkers suggests damage to the kidney proximal tubule at the onset of NE. Also, upregulation of biomarkers of inflammation and leukocyte chemotaxis were detected in NE urine. Furthermore, increased clusterin levels were found in early- and late-phase NE urine. Comparative analysis revealed that clusterin is a biomarker, upregulated in NE urine.

## 1. Introduction

Hemorrhagic fever with renal syndrome (HFRS) is a zoonotic disease caused by several viruses, which are members of the *Hantavirus* genus [1]. The mild form of HFRS, often referred to as nephropathia epidemica (NE), is endemic in the Republic of Tatarstan, Russia [2]. *Puumala* virus, a hantavirus species, is the main causative agent of NE in Tatarstan. NE is acquired by inhaling a virus-contaminated aerosol [3]. The natural reservoir of *Puumala* virus is the bank vole *(Myodes glareolus)*, where infection persists for life without apparent symptoms, whereas infection in humans is characterized by a constellation of clinical symptoms and can sometimes be fatal [4, 5].The fatality rate is about 0.4% with the main cause of death being acute renal failure with disseminated intravascular coagulation (DIC) syndrome.

The dominant clinical feature of NE is kidney insufficiency, which initially presents as back pain.

Soon after, the disease progresses to an oliguric phase, characterized by decreased urine output (<500 mL urine in 1–3 days). Some patients develop anuria, where no urine is produced. The oliguric period is the most critical due to the high likelihood of developing the life-threatening complications. Urinalysis reveals proteinuria and presence of erythrocytes [6]. Additionally, serum levels of creatinine can reach up to 640 *μ*M/L in some patients and could require hemodialysis [7]. Clinical recovery begins with the onset of diuresis [8, 9].

Damage to kidney tissue has been reported in NE cases. Histologically, hantavirus infection is defined as a tubulo-interstitial nephritis with prominent leukocyte infiltration and interstitial hemorrhages [10, 11]. Additionally, IgM, complement component C3, and fibrin deposits along the basal side of the tubular epithelial cells have been reported [11]. Electron microscopy of renal biopsies reveals discrete peritubular capillary damage with signs of capillary leakage and erythrocyte extravasation [7]. Interstitial leukocyte infiltration is composed predominantly of mononuclear immune effectors including CD8+ cytotoxic T lymphocytes and CD68+ macrophages [7]. It has been suggested that infiltrating mononuclear leukocytes play a role in the pathogenesis of kidney tissue damage during hantavirus infection. For example, increased expression of the TNF*α*, TGF*β*, and PDGF has been observed in cells infiltrating the peritubular area of the distal nephron [12]. Also, increased expression of ICAM-1, VCAM, and PECAM in kidney tissue during the infection has been reported. Together, histological data indicate pathological changes to be more prominent in the tubular area of the nephron and that less damage, if any, is detected in the glomerular area [12].

Examination of kidney biopsies obtained from the acute NE cases is an ideal approach to study the mechanisms of renal pathology. However, collection of tissue samples during NE is often not feasible, especially in patients with severely impaired kidney function. Collecting multiple kidney biopsies is impracticable. Therefore, most of our knowledge on kidney tissue damage has come from studying tissue collected postmortem. However, postmortem tissue reflects the late stage of the NE, and therefore, these data have limited value to determine tissue damage at the early stages of the disease or in nonfatal cases.

Clinically, NE is characterized by renal dysfunction which progresses through several stages including oliguric, polyuric, and convalescent stages [13, 14]. Currently, several methods are utilized to evaluate renal function including measurement of creatinine clearance and blood urea nitrogen (BUN). Both methods have limitations. For example, creatinine clearance requires urine collection for over 24 hours, which patients find inconvenient, and therefore, collections are often inaccurate. BUN is a less reliable marker of glomerular filtration rate because it can be affected by factors unrelated to the renal function. It is also important to note that changes in serum creatinine and BUN concentrations primarily indicate presence of changes in filtration capacity and, thus, are not always reflective of tissue injury. Therefore, there is a need for an alternative noninvasive method to assess kidney performance in NE cases.

In this study, we sought to identify biomarkers of kidney tissue injury in NE urine samples. Additionally, we wanted to examine whether urine samples can be used to determine presence of NE damage to specific nephron structures. Analysis of 12 biomarkers suggests damage to the kidney proximal tubule early after the onset of NE. Furthermore, upregulation of biomarkers of inflammation and mononuclear leukocyte chemotaxis were detected in NE urine. Increased clusterin levels indicate injury to kidney tissue at the early phase of the disease. Clusterin levels remained upregulated during the convalescent phase as well, suggesting that kidney tissue damage is still present at the time of hospital discharge. Comparative analysis revealed that clusterin is a biomarker, upregulated in NE urine.

## 2. Materials and Methods

### 2.1. Patients

Urine samples were collected from a total of 64 patients (56 male and 8 female) hospitalized in the Republic Clinical Hospital for the Infectious Disease, Republic of Tatarstan, Russia. All 64 subjects provided at least one urine specimen for the purpose of urine biomarker analysis, and 36 subjects (30 male and 6 female) provided a second urine sample before being discharged from the hospital. Additionally, urine samples were collected from patients diagnosed with gout (32), type 2 diabetes (15), systemic lupus erythematosus (SLE) (8), and chronic kidney insufficiency (CKI) (10). Selection of these diseases was based on the fact that clinical presentation included symptoms of renal dysfunction. Urine samples from 51 (33 male and 18 female) healthy individuals were also collected. The Institutional Review Board of Kazan Federal University approved this study, and informed consent was obtained from each study subject according to the guidelines approved under this protocol (article 20, Federal Law “Protection of Health Right of Citizens of Russian Federation” N323-FZ, November 21, 2011).

Diagnosis of NE was based on clinical presentation and serologically confirmed by detection of antihantavirus antibodies. Two serum samples were collected from each patient. The first sample was collected at the time of admission, and the second sample 7–10 days later. Detection of antihantavirus antibodies was determined by indirect chemiluminescence (Vector, Novosibirsk, Russia). Serum samples were considered positive when the titer of antihantavirus antibody in the second serum sample was ≥4x the first sample.

### 2.2. Multiplex Analysis

Human kidney toxicity panels 1 and 2 (Bio-Rad, Hercules, CA) were used to analyze urine samples according to the manufacturer's recommendations. Kidney toxicity panel 1 detects calbindin, clusterin, glutathione S-transferase-*π* (GST-*π*), IL-18, kidney injury molecule-1 (KIM-1), and monocyte chemotactic protein-1 (MCP-1; CCL2), while kidney toxicity panel 2 detects albumin, beta-2-microglobulin (*β*2M), cystatin C, neutrophil gelatinase-associated lipocalin (NGAL), osteopontin, and trefoil factor 3 (TFF3).

### 2.3. Statistical Analysis

Data are presented as mean ± SE. Statistical analysis was performed using Student's *t*-test for comparisons between individual experimental groups (infected and noninfected). Significance was established at a value of *P* < 0.05.

## 3. Results

### 3.1. Changes in Kidney Toxicity Biomarkers in NE Urine

Changes in renal function were analyzed using the kidney toxicity panels 1 and 2. Urine samples were separated into early (7.8 ± 0.3 days) and late (14.2 ± 0.3) based on the time of collection. Elevated serum creatinine (266.3 ± 18.8 *μ*mol/L) and urea (15.9 ± 0.9 mg/dL) were found in early-phase NE serum as compared to controls (Table 1). Also, oliguria was characteristic of early-phase NE (572.5 ± 34.6 mL/day). At the late-phase NE, creatinine and urea levels decreased, yet still remaining significantly higher than in controls (89.2 ± 1.2 versus 67.1 ± 1.8; *P* < 0.0001). Also, the urine output was higher in late-phase NE cases as compared to controls (2077.3 ± 49.7 versus 1787.8 ± 31.1; *P* < 0.0001). Thirty patients developed polyuria (>2000 mL/day).

Early-phase NE urine samples were characterized by approximately 10 times higher level of clusterin and KIM-1 as compared to those in controls (Table 2). Additionally, upregulation of urine levels of CCL2, IL-18, and TFF3 in the early-phase NE samples was detected as compared to that in controls. Although average values of calbindin in the early-phase NE were higher than in controls, differences were not significant. Levels of *β*2M were lower in NE cases as compared to those in controls. Urine levels of GST-*π*, albumin, cystatin, NGAL, and osteopontin in the early-phase NE urine remained similar to those in controls.

Analysis of the late-phase NE urine samples revealed upregulation of clusterin, KIM-1, IL-18, and CCL2 in NE as compared to those in controls. Similar to that in the early-phase NE urine, levels of calbindin, albumin, NGAL, and osteopontin did not differ from those in controls. However, significant upregulation of GST-*π* and cystatin C was characteristic of late-phase NE urine. Interestingly, TFF3 level declined in the late-phase NE urine, and the level of this biomarker did not differ from that in controls.

### 3.2. Comparative Analysis of Kidney Toxicity Biomarkers in NE, Gout, Type 2 Diabetes, CKI, and SLE

Impaired renal function is not unique to the NE pathogenesis. Therefore, we sought to determine whether the changes found in kidney toxicity biomarkers are specific to NE. Urine samples were collected from patients diagnosed with several diseases known to develop renal pathology including gout, type 2 diabetes, CKI, and SLE. Early- and late-phase NE samples were combined and used to analyze differences in kidney toxicity biomarkers between diseases (Table 2). Upregulation of clusterin was detected in NE and SLE cases. Interestingly, urine levels of GST-*π* were downregulated in all cases except NE. However, only in gout was GST-*π* significantly lower than that in controls. Comparison of urinary IL-18 levels revealed a significantly high level of this cytokine in NE and SLE cases. Interestingly, IL-18 levels were significantly higher in SLE cases as compared to NE, suggesting that inflammatory reaction plays a greater role in SLE renal pathology. Urinary levels of KIM-1 were universally upregulated in all samples studied, suggesting that KIM-1 levels reflect changes in renal function that are not disease specific. CCL2 levels were increased in urine samples of NE, type 2 diabetes, CKI, and SLE (Table 3). Urinary levels of CCL2 in gout were upregulated as well, though they did not differ significantly from those in controls. Upregulated CCL2 levels suggest the presence of an inflammatory reaction and chemotaxis of the mononuclear leukocytes.

## 4. Discussion

Severity of the disease and development of complications in NE heavily depend on impairment of renal function. Clinically, disease progression has been monitored using laboratory findings reflecting renal performance including BUN and creatinine levels. For example, the severe form of NE is characterized by high BUN and creatinine levels: BUN 20 mM/L and creatinine up to 600 *μ*M/L [15–17]. The moderate form of the disease has similar symptoms that are subtler, with levels of BUN and creatinine over 19 mM/L and 200–300 mM/L, respectively. Finally, the mild form presents with minimal changes in renal function and often remains undiagnosed [15–17]. Changes in the urine output are characteristic of oliguria (<500 mL of urine for 1–3 days) and polyuria periods of NE. Most of our knowledge on the NE kidney pathology is based on data from studying postmortem renal tissue.

Although valuable for understanding the overall kidney pathology, postmortem data are more relevant to changes in kidney tissue during the late stage of the severe form of the disease. Therefore, pathological changes in renal tissue during the early stages of the disease and in mild or moderate cases remain largely unknown.

Urine is a valuable source of kidney function study. Recently, novel urine biomarkers were introduced to evaluate renal function. For example, NGAL has been suggested as a potential biomarker for ischemic or nephrotoxic kidney injury [18]. Another biomarker is KIM-1, which has been shown to be upregulated in patients with acute kidney injury as compared to those with chronic kidney disease [19]. These biomarkers can be used to distinguish the site of damage in the nephron and, to some extent, determine whether damage is associated with inflammation, leukocyte migration, immune complex (IC) deposition, and so on. Despite extensive research, little is known about the pathological changes in the NE kidney.

Therefore, we analyzed urine samples collected from the NE cases to identify the site of nephron damage. Also, we sought to reveal biomarkers in the NE urine with a potential diagnostic value. Analysis of the urinary level of 12 biomarkers suggests a strong inflammatory milieu in NE kidney tissue. For example, early upregulation of IL-18 was detected in early- and late-phase NE urine. IL-18 is associated with the severe inflammatory reactions. Study conducted by Carson and colleagues have demonstrated that combination of IL-18 and IL-12 can result in a systemic inflammation leading to a 100% animal mortality [20]. Our finding of an increased urinary level of clustering also suggests an early inflammatory milieu in NE renal tissue. Clusterin expression is absent in normal kidney; however, its expression is often upregulated in injured renal tissue [21, 22]. Expression of clusterin is restricted to epithelial cells and often detected in tubular cells of injured kidneys [23, 24]. It has been demonstrated that only cytoplasmic and secreted forms of clusterin are protective [25] while its nuclear form is proapoptotic [26]. Studies have shown that clusterin can bind to immunoglobulins and complement components [27, 28]. Increased deposition of complement and immunoglobulin is consistently reported in NE renal tissue collected postmortem. It should be noted that clusterin plays a protective role in renal tissue, as it has been shown to prevent formation of complement attacking complex, and inhibits apoptosis and matrix metalloproteases activation. Our data suggest that upregulation of clusterin could account for deposition of complement components and immunoglobulin in NE cases. However, clusterin may function to protect renal tissue from the damaging effects of complement. For example, it has been shown that clusterin can control the lytic activity of membrane attack complex (MAC) by binding to C5b-7, thus preventing its binding to cell membranes [29, 30]. Also, it has been suggested that clusterin could interfere with formation of terminal complement components, while not affecting glomerular antibody binding and C3 deposition [31]. Our finding of upregulation of clusterin in NE urine implicates its role in disease pathogenesis. More study is required to evaluate whether clusterin plays a protective role in NE. An increased level of KIM-1 was detected in NE urine samples, suggesting damage to the proximal tubule. Normally undetectable, KIM-1 is induced more than any other proteins when kidneys are injured. Studies have shown that KIM-1 upregulation exceeding 12-folds and can be associated with the risk of development of acute tubular necrosis [19]. KIM-1 is found to be localized to the apical surface of surviving proximal tubule epithelial cells [32]. Studies have suggested that KIM-1 could be used as biomarkers for acute renal failure. The diagnostic value of KIM-1 as a predictor of acute kidney failure has been demonstrated by Vaidya et al. [33], and it was further confirmed by Liangos et al. [34]. Severe cases of NE are characterized by acute kidney failure [35]; however, the clinical signs of this life-threatening complication appear during late stages of the disease. Therefore, identifying molecules which could serve as early biomarkers of acute kidney failure has high clinical importance. More study is required to further validate KIM-1 as biomarkers of an acute kidney failure in NE. Analysis of kidney toxicity biomarkers collectively suggests that tubular injury is a hallmark of NE. It appears that the injury to tubular epithelium could lead to functional impairment. This assumption is based on the fact that two biomarkers of tubular function differed significantly in the NE and control urine. Levels of *β*2M were lower in urine of NE cases. *β*2M is a molecule which is readily filtered through the glomerulus and almost completely reabsorbed and destroyed by proximal tubular cells [36].

Additionally, we found another marker of proximal tubular dysfunction, cystatin C, to be changed in NE urine. Together, our data suggest that tubular injury in NE cases leads to disruption of the tubular function in the proximal nephron.

Significant upregulation of GST-*π* has been found in NE urine samples collected during the convalescent phase. GST-*π* is constitutively expressed in distal nephron epithelium, and it is released into the urine upon epithelial cell damage [37, 38], suggesting the injury to the distal nephron epithelium. Since upregulation of GST-*π* in urine was found only in the late-phase NE samples, we conclude that abnormalities in kidney function at the end of the disease could be related to the distal nephron epithelial damage. Therefore, we suggest that damage to the proximal epithelium determines the early changes in kidney function, while injury to proximal and distal epithelia causes the symptoms of renal insufficiency at late stages of the disease.

In conclusion, we identified clusterin as a biomarker upregulated in urine of NE and SLE cases. Both diseases are characterized by increased serum level of IC and complement activation. Therefore, we suggest that immune mechanisms are playing a role in the pathogenesis of kidney damage in NE, where IC and complement deposition are essential. Clusterin was found to be upregulated in the early as well as convalescent phases of the disease, suggesting prolonged kidney injury extending beyond the recovery stage. Clusterin could account for deposition of complement components and immunoglobulin in NE kidneys; however, its role in pathogenesis of renal injury remains to be determined.

## Figures and Tables

**Table 1:** Laboratory characteristics of NE cases.

Variables	Control	NE1	NE2	*P* value
Serum creatinine (*μ*mol/dL)	67.1 ± 1.8	266.3 ± 18.8	89.2 ± 1.2	^∗^ *P* < 0.0001 ^∗∗^*P* < 0.0001
Serum urea (mg/dL)	4.5 ± 0.2	15.9 ± 0.9	7.2 ± 0.2	^∗^ *P* < 0.0001 ^∗∗^*P* < 0.0001
Total urine output (mL/day)	1787.8 ± 31.1	572.5 ± 34.6	2077.3 ± 49.7	^∗^ *P* < 0.0001 ^∗∗^*P* < 0.0001
Hantavirus antibody (titer)	458 ± 24.0	824 ± 120.3		

$\begin{array}{c} {}^{*}P \end{array}$ values as compared to those of control. ^∗∗^*P* values as compared between NE1 and NE2 groups.

**Table 2:** Changes in kidney toxicity markers in NE urine.

	Control	NE1	NE2
Calbindin	66459.1 ± 25284.1	175672.7 ± 78552.9	87961.2 ± 30921.1
Clusterin	19785.8 ± 6384.3	114057.5 ± 61050.4 *P* < 0.008	193594.6 ± 113924.7 *P* < 0.002
GST-*π*	33688.5 ± 10201.6	46521.7 ± 31162.4	125749.1 ± 65870.5 *P* < 0.02
IL-18	5.8 ± 0.6	38.1 ± 11.3 *P* < 0.0001	34.1 ± 9.7 *P* < 0.0001
KIM-1	80.8 ± 10.9	905.0 ± 275.9 *P* < 0.0001	709.9 ± 189.3 *P* < 0.0001
CCL2	70.6 ± 16.4	457.7 ± 148.8 *P* < 0.0001	404.2 ± 109.3 *P* < 0.0001
Albumin	1513.4 ± 97.5	1437.9 ± 66.8	1681.4 ± 340.5
*β*2M	29.7 ± 3.1	15.8 ± 1.3 *P* < 0.0001	19.0 ± 1.9 *P* < 0.02
Cystatin C	18.8 ± 6.4	440.3 ± 198.8	72.5 ± 21.1 *P* < 0.01
NGAL	17.5 ± 3.9	34.9 ± 3.4 *P* < 0.002	31.7 ± 4.9 *P* < 0.03
Osteopontin	131.6 ± 17.9	279.7 ± 42.5 *P* < 0.01	140.6 ± 39.2
TFF3	121.5 ± 7.5	138.5 ± 5.7	147.1 ± 6.8

NE1: first urine sample; NE2: second urine sample.

**Table 3:** Comparative analysis of kidney toxicity markers in NE, SLE, type 2 diabetes, CKI, and gout.

Patient group	Calbindin	Clusterin	GST-*π*	IL-18	KIM-1	CCL2
Control	66459.1 ± 25284.1	19785.8 ± 6384.3	33688.5 ± 10201.6	5.8 ± 0.6	80.8 ± 10.9	70.6 ± 16.4
NE	85876.1 ± 2689.2 *P* < 0.02	174341.9 ± 72065 *P* < 0.03	47344.4 ± 24993	32.7 ± 8.6 *P* < 0.002	813.2 ± 205.2 *P* < 0.0002	442.7 ± 100.6 *P* < 0.0005
Gout	22727.9 ± 4971.2	23793.4 ± 12298.9	3511.8 ± 646.9 *P* < 0.04	10.7 ± 2.1	236.5 ± 77.8 *P* < 0.008	144.3 ± 49.1
Type 2 diabetes	34102.5 ± 10407.8	51272.1 ± 22931.1	14008.6 ± 3865.4	10.8 ± 2.9	330.5 ± 118.4 *P* < 0.0002	412.5 ± 145.1 *P* < 0.0001
CKI	28778.8 ± 10907.8	49218.5 ± 27304.5	14846.8 ± 3772.1	14.6 ± 4.4	414.4 ± 186.9 *P* < 0.0001	526.9 ± 409.4 *P* < 0.0001
SLE	71437.9 ± 42320.5	66160.7 ± 27984.5 *P* < 0.03	27211.1 ± 6190.7	636.3 ± 622.3 *P* < 0.01	526.5 ± 308.9 *P* < 0.0002	336.7 ± 148.4 *P* < 0.0003

## References

[1] Mustonen J., Mäkelä S., Outinen T. (2013). The pathogenesis of nephropathia epidemica: new knowledge and unanswered questions. *Antiviral Research*.

[2] Li J., Chen J., Yang G. (2015). Case-control study of risk factors for human infection with avian influenza A (H7N9) virus in Shanghai, China, 2013. *Epidemiology and Infection*.

[3] Garanina S. B., Platonov A. E., Zhuravlev V. I. (2009). Genetic diversity and geographic distribution of hantaviruses in Russia. *Zoonoses and Public Health*.

[4] Yanagihara R., Amyx H. L., Gajdusek D. C. (1985). Experimental infection with *Puumala* virus, the etiologic agent of nephropathia epidemica, in bank voles (*Clethrionomys glareolus*). *Journal of Virology*.

[5] Diglisic G., Rossi C. A., Doti A., Walshe D. K. (1999). Seroprevalence study of *Hantavirus* infection in the community based population. *Maryland Medical Journal*.

[6] Khaiboullina S. F., Martynova E. V., Khamidullina Z. L. (2014). Upregulation of IFN-*γ* and IL-12 is associated with a milder form of hantavirus hemorrhagic fever with renal syndrome. *European Journal of Clinical Microbiology & Infectious Diseases*.

[7] Ferluga D., Vizjak A. (2008). Hantavirus nephropathy. *Journal of the American Society of Nephrology*.

[8] Bi Z., Formenty P. B., Roth C. E. (2008). Hantavirus infection: a review and global update. *Journal of Infection in Developing Countries*.

[9] Cosgriff T. M. (1991). Mechanisms of disease in *Hantavirus* infection: pathophysiology of hemorrhagic fever with renal syndrome. *Reviews of Infectious Diseases*.

[10] Mustonen J., Helin H., Pietilä K. (1994). Renal biopsy findings and clinicopathologic correlations in nephropathia epidemica. *Clinical Nephrology*.

[11] Groen J., Bruijn J. A., Gerding M. N., Jordans J. G., Moll van Charante A., Osterhaus A. D. (1996). Hantavirus antigen detection in kidney biopsies from patients with nephropathia epidemica. *Clinical Nephrology*.

[12] Temonen M., Mustonen J., Helin H., Pasternack A., Vaheri A., Holthöfer H. (1996). Cytokines, adhesion molecules, and cellular infiltration in nephropathia epidemica kidneys: an immunohistochemical study. *Clinical Immunology and Immunopathology*.

[13] Bren A. F., PavlovČIČ S. K., Koselj M., KovaČ J., Kandus A., Kveder R. (1996). Acute renal failure due to hemorrhagic fever with renal syndrome. *Renal Failure*.

[14] Makela S., Ala-Houhala I., Mustonen J. (2000). Renal function and blood pressure five years after *Puumala* virus-induced nephropathy. *Kidney International*.

[15] Turcinov D., Puljiz I., Markotić A., Kuzman I., Begovac J. (2013). Clinical and laboratory findings in patients with oliguric and non-oliguric hantavirus haemorrhagic fever with renal syndrome: an analysis of 128 patients. *Clinical Microbiology and Infection*.

[16] Germash E. I., Timokhov V. S., Zagidullin ShZ, Zagidullin I. M., Ozhgikhin S. N. (1997). The pathogenetic therapy of patients with a severe form of hemorrhagic fever and acute kidney failure. *Terapevticheskiĭ Arkhiv*.

[17] Tsai T. F. (1987). Hemorrhagic fever with renal syndrome: clinical aspects. *Laboratory Animal Science*.

[18] Mishra J., Ma Q., Prada A. (2003). Identification of neutrophil gelatinase-associated lipocalin as a novel early urinary biomarker for ischemic renal injury. *Journal of the American Society of Nephrology*.

[19] Han W. K., Bailly V., Abichandani R., Thadhani R., Bonventre J. V. (2002). Kidney injury molecule-1 (KIM-1): a novel biomarker for human renal proximal tubule injury. *Kidney International*.

[20] Carson W. E., Dierksheide J. E., Jabbour S. (2000). Coadministration of interleukin-18 and interleukin-12 induces a fatal inflammatory response in mice: critical role of natural killer cell interferon-gamma production and STAT-mediated signal transduction. *Blood*.

[21] Dvergsten J., Manivel J. C., Correa-Rotter R., Rosenberg M. E. (1994). Expression of clusterin in human renal diseases. *Kidney International*.

[22] Rosenberg M. E., Paller M. S. (1991). Differential gene expression in the recovery from ischemic renal injury. *Kidney International*.

[23] Harding M. A., Chadwick L. J., Gattone V. H., Calvet J. P. (1991). The SGP-2 gene is developmentally regulated in the mouse kidney and abnormally expressed in collecting duct cysts in polycystic kidney disease. *Developmental Biology*.

[24] Correa-Rotter R., Ibarra-Rubio M. E., Schwochau G. (1998). Induction of clusterin in tubules of nephrotic rats. *Journal of the American Society of Nephrology*.

[25] Jung G. S., Kim M. K., Jung Y. A. (2012). Clusterin attenuates the development of renal fibrosis. *Journal of the American Society of Nephrology*.

[26] Kim N., Yoo J. C., Han J. Y. (2012). Human nuclear clusterin mediates apoptosis by interacting with Bcl-XL through C-terminal coiled coil domain. *Journal of Cellular Physiology*.

[27] Wilson M. R., Easterbrook-Smith S. B. (1992). Clusterin binds by a multivalent mechanism to the Fc and Fab regions of IgG. *Biochimica et Biophysica Acta (BBA) - Protein Structure and Molecular Enzymology*.

[28] French L. E., Tschopp J., Schifferli J. A. (1992). Clusterin in renal tissue: preferential localization with the terminal complement complex and immunoglobulin deposits in glomeruli. *Clinical and Experimental Immunology*.

[29] Choi N. H., Mazda T., Tomita M. (1989). A serum protein SP40,40 modulates the formation of membrane attack complex of complement on erythrocytes. *Molecular Immunology*.

[30] Choi N. H., Nakano Y., Tobe T., Mazda T., Tomita M. (1990). Incorporation of SP-40,40 into the soluble membrane attack complex (SMAC, SC5b-9) of complement. *International Immunology*.

[31] Saunders J. R., Aminian A., McRae J. L., O'Farrell K. A., Adam W. R., Murphy B. F. (1994). Clusterin depletion enhances immune glomerular injury in the isolated perfused kidney. *Kidney International*.

[32] Ichimura T., Bonventre J. V., Bailly V. (1998). Kidney injury molecule-1 (KIM-1), a putative epithelial cell adhesion molecule containing a novel immunoglobulin domain, is up-regulated in renal cells after injury. *The Journal of Biological Chemistry*.

[33] Vaidya V. S., Waikar S. S., Ferguson M. A. (2008). Urinary biomarkers for sensitive and specific detection of acute kidney injury in humans. *Clinical and Translational Science*.

[34] Liangos O., Perianayagam M. C., Vaidya V. S. (2007). Urinary *N*-acetyl-beta-(d)-glucosaminidase activity and kidney injury molecule-1 level are associated with adverse outcomes in acute renal failure. *Journal of the American Society of Nephrology*.

[35] Rasche F. M., Uhel B., Ulrich R. (2004). Thrombocytopenia and acute renal failure in *Puumala* hantavirus infections. *Emerging Infectious Diseases*.

[36] Bernier G. M., Conrad M. E. (1969). Catabolism of human beta-2-microglobulin by the rat kidney. *American Journal of Physiology-Legacy Content*.

[37] Sundberg A., Appelkvist E. L., Dallner G., Nilsson R. (1994). Glutathione transferases in the urine: sensitive methods for detection of kidney damage induced by nephrotoxic agents in humans. *Environmental Health Perspectives*.

[38] Harrison D. J., Kharbanda R., Cunningham D. S., McLellan L. I., Hayes J. D. (1989). Distribution of glutathione S-transferase isoenzymes in human kidney: basis for possible markers of renal injury. *Journal of Clinical Pathology*.

